# Automated mapping of electronic data capture fields to SDTM

**DOI:** 10.1371/journal.pone.0312721

**Published:** 2024-11-07

**Authors:** Eric Yang, Laura Katz, Sushila Shenoy

**Affiliations:** Medidata Solutions, New York, New York, United States of America; Khalifa University, UNITED ARAB EMIRATES

## Abstract

**Objective:**

The goal of this work is to reduce the amount of manual work required to go from data capture to regulatory submission. It will be shown that the use of Siamese networks will allow for the generation of embeddings that can be used by traditional machine learning classifiers to perform the classification at much higher levels of accuracy than standard approaches.

**Methods:**

Siamese networks are a method for training data embeddings such that data within the same class are closer with respect to a given distance metric than they are to data points in another class. Because they are designed to learn similarity within pairs of data points, they work well in situations where the number of classes is relatively large compared to the number of training samples. In this work, we will show that embeddings generated via a Siamese network from metadata associated with electronic data capture forms can be used to predict the associated SDTM field.

**Results:**

With a relatively simple network coupled with a basic classification algorithm, the proposed method can achieve accuracies greater than 90%, which is significantly higher than what has been achieved with traditional methods, with many of the inaccurate mappings due to a lack of training data. In many cases, there is a 15% increase in accuracy vs. more traditional methods.

**Conclusion:**

Leveraging Siamese networks, it is possible to generate embeddings that efficiently represent data fields in a lower dimensional space. This allows the creation of a system that can automatically map between data schemas at high levels of accuracy. Such systems represent the first step in automating one of the many labor-intensive data management tasks associated with clinical trials.

## Introduction

One of the less heralded advances in clinical data management has been the standardization of trial data into the Standard Data Tabulation Model (SDTM) [1]. The drive to standardize the data has led to efficiencies in the regulatory process, as well as allowed for the streamlined comparison between trials after the trial has been completed [2,3]. However, while significant efficiency gains have been observed post-submission, the burden of work to perform this operation is still substantial [4]. While it is a net win to spread the work amongst a larger set of organizations, the total amount of work required to standardize a dataset to SDTM remains. The primary challenge is that oftentimes the electronic data capture systems (EDC) are designed for ease of trial conduct and the mapping to SDTM remains a secondary priority. The current process of standardizing data from EDC systems to SDTM generally requires the engagement of programmers who are responsible for reading the annotated case report form (CRF) and writing scripts to convert them into the SDTM format. As it currently stands, this process is heavily labor intensive, and in many cases non-repeatable [5].

Going forward, there are two solutions generally considered. The first is to better standardize EDC builds [3]. Essentially, once a mapping for a trial exists, all future trials will follow the same pattern. For instance, the CDISC organization has defined a standard for EDC builds that hopefully will make the final mapping process more streamlined [6]. Traditionally however, there has been resistance against standardization given that the primary focus of EDC builds has been ease of use for the clinical sites and/or the desire to design forms that are closer to how the data is expected to be collected by the clinical site rather than how the data is going to be analyzed [7].

The second solution is to determine whether ML methods can aid in the mapping process so that the mapping process can be made more efficient. While standardization is the most straightforward approach, it has certain limitations such as the loss of flexibility, the difficulty in establishing standards in general, and most importantly, the inability to map legacy trials that have not been submitted previously in SDTM format. If it were possible to map legacy trials to SDTM format at scale, it would greatly increase the value of the legacy data by allowing for cross-trial analysis without requiring the overhead of manually harmonizing the datasets prior to analysis. Most solutions to the SDTM mapping problem revolve around the use of workflow tools, or the reuse of forms to minimize the amount of repeated work that has to be done [8]. A search through Google has identified only a single ML-based technique for doing this SDTM Mapper by Sam Tomioka (https://github.com/stomioka/sdtm_mapper).

One of the reasons that we believe that an automated mapping process is feasible is because even without a written specification as to which field maps to which SDTM variable, humans can manually curate and map the data from various pieces of metadata that describe the field itself. This suggests to us that there exists sufficient information within the metadata to inform the mapping process and that an ML model should be feasible. However, while there is an acknowledgment that this process should be possible, there has been comparatively little work done on the topic, with one of the primary barriers to building a workable prototype being the relative lack of training data, especially when compared to the number of fields that require mapping [9]. Even the largest pharmaceutical companies will have only a few thousand clinical trials from which to leverage for training the algorithm [10]. However, developments in few-shot learning have led us to believe that the conditions are sufficient to automate the mapping process between EDC systems and SDTM.

## Method

Briefly, we propose using Siamese networks to generate EDC metadata-based embeddings and then utilizing a traditional ML classifier on these embeddings to predict the associated SDTM field. A Siamese network consists of twin subnetworks, i.e. networks with a shared architecture and shared weights. It is trained in such a way that similar data points will have a lower dimensional embedding that is closer based on some distance metric, such as the Euclidean distance, than dissimilar data points [11]. This allows us to train a subnetwork in which the lower dimensional embedding of each field can be compared to all the other fields. Given this paradigm, this allows us to take N data points, and expand them to O(N^2^) pairs from which to train the neural network, thus providing one avenue by which to deal with the lack of data available for training [12]. After obtaining this embedding, the lower dimensional representation can then be used to generate a final predictive model to classify the form fields to their SDTM domains.

**Fig 1:**
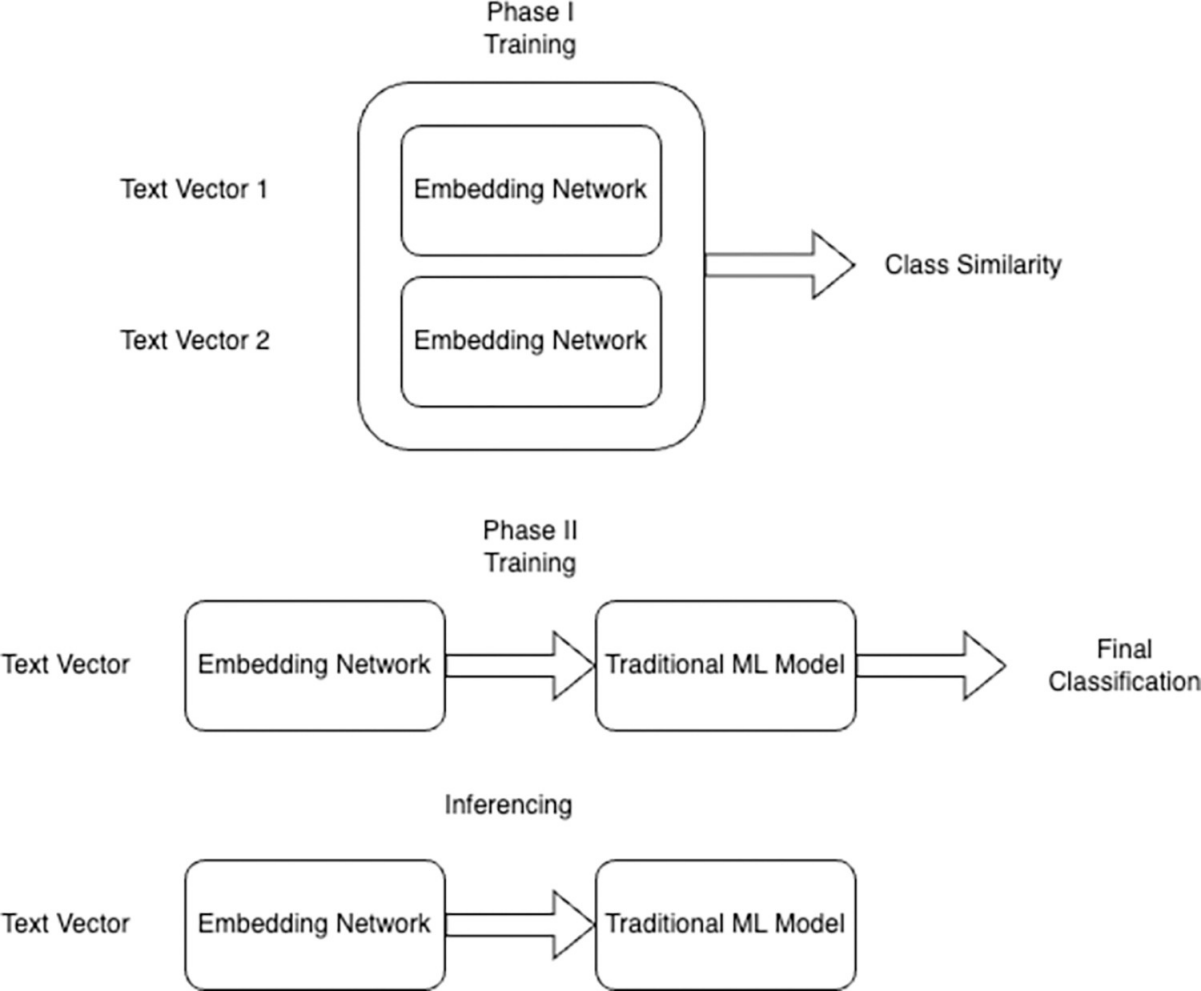
The overall process by which the Siamese model is trained and used for prediction of final classification of SDTM fields.

In this specific implementation, the Siamese network will calculate a 32-dimensional embedding such that Rave entries that map to the same SDTM field will have a distance approaching 0, and pairs of Rave entries that map to different fields will have a distance approaching 1. To make this embedding model useful for direct prediction, this embedding network is then combined with another classifier to perform the final prediction. In the context of this work, a k-NN model is just an illustrative model meant to satisfy an auto-suggest use case in which the auto-suggest box could be populated with majority vote, closest neighbor, and whether the appropriate choice was found in the top N. The overall schematic of the prediction process is shown in **Fig 1.**

### Data

The underlying data that is being used is the metadata associated with the electronic case report forms (eCRF). These consist of the Form Name (FormOID), the pre-text (description of the field in question), the post-text (additional description such as the format that the input is expected to take such as expected date format), and the FieldOID which is the internal identifier for the specific field in question. These four fields of information are then associated with an SDTM field. For the sake of this evaluation, the dataset was limited to the following domains: Adverse Events (AE), Concomitant Medications (CM), Medical History (MH), Vital Signs (VS), and Demographic (DM) domains. The fields associated with these domains were manually mapped to SDTM by human curators. While this does not represent a comprehensive list of domains that are part of an SDTM dataset, these domains are a reasonable representation of the structures that need to be mapped.

Normally, domains in SDTM format are represented as a mix of unpivoted (skinny) and pivoted (wide) formats, for simplification, every domain was represented as an unpivoted table. In our opinion, this does not represent a significant shortcoming of the work, because there is a straightforward way of converting a pivoted to an un-pivoted representation, and this is known based on the SDTM standard. Additionally, there was no attempt to map composite fields (fields that are the result of mathematical operations between one or more fields such as BMI). In the training data, there were ~7000 separate entries that correspond to 43 different SDTM fields. A sample of the metadata associated with the EDC systems is given in **Table 1.**

**Table 1:** A sample of the data that is used as features for the Siamese network.

FormOID	FieldOID	PreText	PostText
DRUG_ADMINISTRATION	AGT_ACTL_DOSE	Actual Total Dose	(ddddd.ddd)
VITAL_SIGNS	DIAST_BP_VAL	Diastolic Blood Pressure Value	(dddddddd)
RAND	RNDDAT	**Randomization Date**	(GMT)
ELIG	ELIGBRTHDAT	Date of Birth:	(MM/DD/YYYY)
DC	DCSYSBP	Systolic blood pressure:	(mmHg)
EXPIRATION	DEATH_MDY_DT	**Date of Death**	(MMM dd yyyy)

To preprocess the input into a format usable by a neural network the text is first converted to raw text by stripping out any HTML tags that may be present within the text. This was done with the Python html2text library. After conversion, the four fields are concatenated together. While there are undoubtedly more sophisticated methods to combine the different fields, the simplest method was used to determine what the baseline performance of the overall method would be. The maximum length of this string was 1827 words. The long string was then dictionary-encoded via Gensim. Dictionary encoding is the process when the first word observed in the text is encoded as 1, the second word observed in the series is encoded as 2, and so forth. This gives us a numerical vector that can be processed by the neural network. In the case where a word is not found in this encoding library, the missing word is encoded as a 0. This is similar to how other systems handle out of vocabulary tokens. While this is not ideal, we elected to evaluate the simple case given that many of the out of vocabulary words came in the FormOID/FieldOID fields which in many cases did not necessarily have sematic meaning.

For the Siamese network, pairs are randomly generated. For each row in the SDTM data 20 pairs are generated where two entries are within the same class and at least 20 pairs are generated where the two entries are associated with two different SDTM fields. The output variable for the Siamese network is 0 if they belong in the same class and 1 if they are not. This mapping operation sets a minimum distance between two entries from the same class, and if they are not in the same class, to set it at some arbitrary distance. To generate the holdout set, the dataset was first stratified by SDTM fields before generating the holdout set, so there was the same proportion of SDTM fields in the training/testing set. The training and testing set were generated via 5-fold cross-validation. Data was split prior to the pairs being generated to make sure that there wasn’t any data leakage from the pairs.

### Model

**Fig 2:**
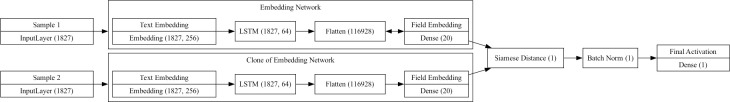
The overall network architecture of the Siamese network utilized in this manuscript.

A Siamese network is a type of neural network architecture that is designed to learn the similarity or dissimilarity between two different inputs. For each pair of training data, the distance is calculated as given in **Eq 1.** It does this by training a neural network that generates an embedding for a given input and then comparing the embeddings between two inputs. In our case, fields that map to the SDTM domain should be more similar than fields that map to different SDTM fields. In a practical sense, two networks that share the same architecture and weights are generated and then merged with an external layer that calculates the distance between the embeddings. In this implementation, the Euclidean distance was chosen. The overall architecture is given in **Fig 2**.


$$d(x,y)=\{\begin{array}{l} 0\;\mathrm{if}\;x\;\mathrm{and}\;y\;\mathrm{are}\;\mathrm{in}\;\mathrm{the}\;\mathrm{same}\;\mathrm{class}\\ 1\;\mathrm{if}\;x\;\mathrm{and}\;y\;\mathrm{do}\;\mathrm{not}\;\mathrm{belong}\;\mathrm{in}\;\mathrm{the}\;\mathrm{same}\;\mathrm{class} \end{array}$$ (1)

The embedding network architecture consisted of a Text Embedding layer, an LSTM Layer with 64 nodes, a Flatten Layer, and a Dense layer with 32 nodes. The text embedding layer was chosen to map the vocabulary to 256 dimensions. These were the initial parameters chosen based on prior experience as working well in a general case and not specifically tuned for this network. These were initially chosen for feasibility before a more computationally intensive hyperparameter search was conducted. However, given the performance of the model with default parameters, hyperparameter tuning was not performed.

The Siamese Distance uses the standard Euclidean distance to calculate the similarity between the two embeddings. This is the standard distance equation and given in **Eq 2.**


$$\sqrt{\overset{32}{\underset{i=0}{\sum }}(x_{i}-y_{i})}$$ (2)

The two layers that require an activation function, namely the LSTM layer and the Final Activation layer utilized a tanh and sigmoid activation function respectively. The tanh activation function was chosen for the LSTM layer due to the desire for the embeddings to be centered around 0 and have a roughly equal distribution of positive and negative values, whereas the final activation function was selected as a sigmoid because of the range of our output variables (0 and 1). Rather than utilizing a pre-trained embedding model such as Bert or Glove [13], we elected to train a bespoke embedding layer as part of this process. This was done because, in prior experience, many of the entries in the data, specifically the FieldOID, FormOID, and the PostText are not found in the public training corpus, and thus generate embeddings that are not semantically related. One of the benefits of utilizing a custom embedding layer is that it’s relatively trivial to switch from a word-based encoding in which unknown words have to be imputed, it is relatively trivial convert to a character based embedding method. Thus, rather than dictionary encoding the individual works, it’s possible to change the input layer to the number of characters x 26 rather than just the number of words.

Note, that other activation functions could have been evaluated but were not because initial experiments suggested that the network was reaching the limits of accuracy based on the size of the training set. The benefits of utilizing a Siamese network are twofold. The first benefit is that because it operates on pairs of data points, given N data samples, the training can be conducted on N^2^ pairs thus greatly increasing the number of training samples that can be used to refine the network. Secondly, depending on the choice of the distance metric, the embeddings can be made to be compact [14] and amenable to the use of secondary classification models.

The subnetworks themselves consist of an embedding layer, an LSTM layer, and a single dense layer. It was decided that the embedding layer would be trained along with the network, rather than utilizing a pre-trained word embedding [15]. The primary reason for this was due to the amount of vocabulary that would be specific to the task such as the FormOIDs and the FieldOIDs, along with the jargon and abbreviations given in the text description, which would make it a poor fit for embeddings trained on more general vocabularies. An embedding layer converts a linear vector of text that has been dictionary-encoded into a matrix with one row per element in the original vector. Words that are found in the same context are then encoded in an N-dimensional vector such that similar words will map to similar vectors. After encoding through an embedding layer, it is then fed through an LSTM network in a way that is similar to how most time series data would be handled. The subnetwork aims to create an embedding of 20 dimensions, which can then be used with other classifiers to do the final classification. For visualization purposes, this 20-dimensional embedding will be reduced to 2 dimensions via PCA [16]. Training was run for 3 epochs with a batch size of 64.

### Final classification

Once the embedding network has been trained via the Siamese network, it can be used to generate low-dimensional embeddings of the text data. From here, standard ML techniques can be used to create a classifier that takes the embedding and generates the final prediction. A kNN classifier [17] was chosen as the final classifier. While other techniques such as XgBoost, Random Forest, or even another neural network could have been used [18], the selection of the kNN model was done because it allowed for additional interrogation as to the quality of the embedding [19]. Essentially the evaluation sought to answer the following question; “Of the N nearest neighbors, how many belong to the same class?” In our specific case, we chose K = 5. This question can be broken down further into 3 questions:

- Is the correct class found within any of the nearest k-neighbors?
- Is the correct class predicted by the nearest neighbor?
- Is the correct class predicted by the majority vote of the k nearest neighbors?

The K-nearest neighbor classifier will return the 5 closest SDTM fields in the training set, and from these 5 classes, we will calculate whether at least one of the classes is correct, whether the closest neighbor is of the proper class, and whether majority votes will arrive at the proper class.

It is important to note that this final classifier was chosen primarily as a way to interrogate the quality of the embedding rather than trying to obtain the best baseline performance, and thus the reported accuracy of the network given below can be simply improved by utilizing a more sophisticated final classifier.

The evaluation of the final classification results was done on a 20% holdout. This allowed us to guarantee that each class in the dataset would show up in both the training and testing set. This work was evaluated by calculating the basic accuracy i.e. the number of predictions in the holdout set that matched their SDTM field, as well as the Macro F1 scores.

### Comparison

As a comparison, two prior methods that were implemented at Medidata for the same task were evaluated vs. the proposed method. These two methods are generally representative of ML approaches for data classification, one using traditional tree-based models and the other using deep learning with pre-trained text embedding. The first implementation utilized XGBoost on the same field metadata, where the metadata was encoded via TF-IDF [20]. The second method was a straightforward deep learning implementation that consisted of a pre-trained text embedding layer (RoBERTa) [21] along with two bi-directional LSTMs used to predict the final class. This NN implementation was done in Keras. Despite being open source, the evaluation of sdtm-mapper was not shown here. That implementation required different data fields than we had available to us. However, the NN implementation that we had was designed to replicate a similar architecture.

Briefly, the first model which utilized TD-IDF + XgBoost, default parameters were used for XgBoost. For TF-IDF, the minimum number of times a word had to be present in the corpus was set at 2. Additional hyperparameter tuning was explored, but not implemented in the reported model because we saw large swings in the testing accuracy between runs, whereas the default parameters were significantly more stable. The Roberta implementation was based on the HuggingFace library [22]. The batch size in this model was selected as 48, and the maximum length of the input text was capped at 100 words. This was trained for 9 epochs. Other parameters were left as the default.

Evaluations were done via the Overall Accuracy which is simply the number of entries that were correctly predicted. However, given that the examples in the SDTM fields are different, we also evaluated the Macro F1 score This weights the accuracy on rare classes as heavily as those in more populated classes, thus preventing highly prevalent fields in our training data from skewing the results [23].

## Results

**Fig 3:**
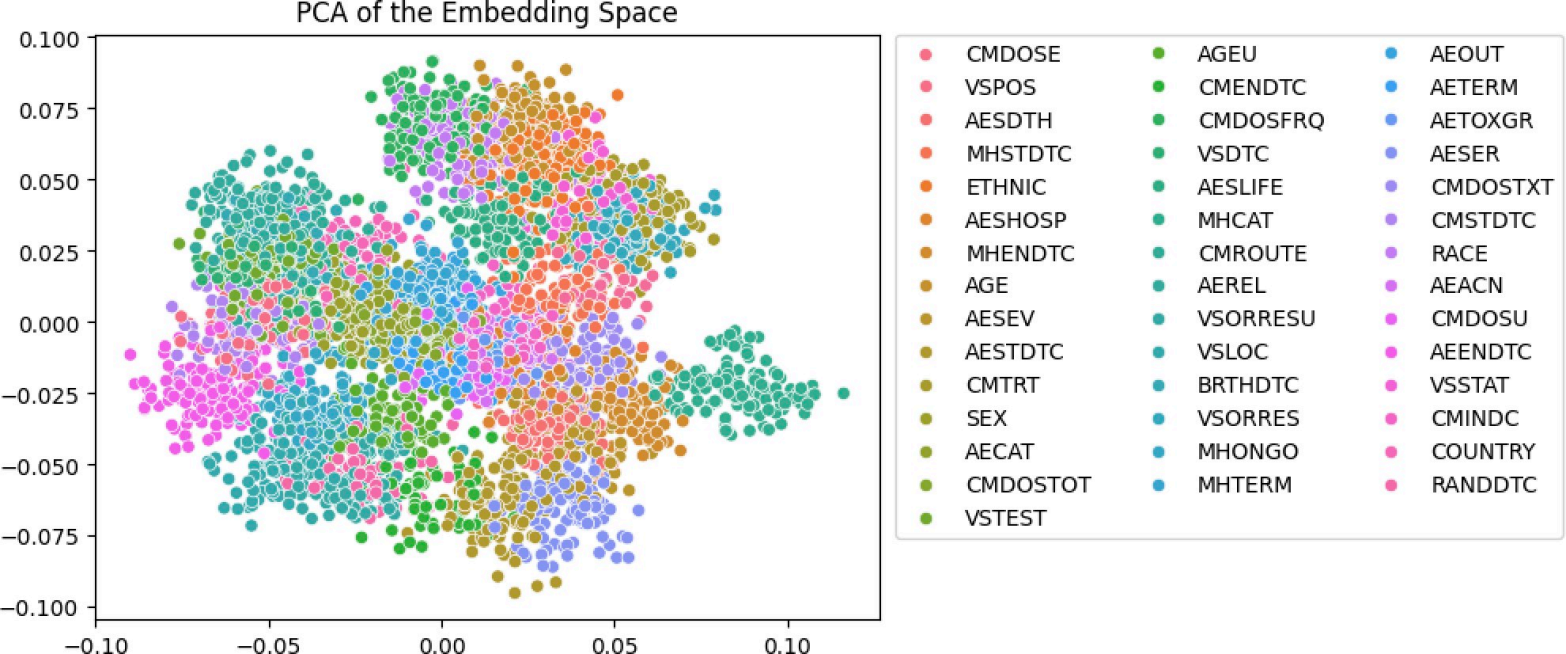
The embedding of the different classes as generated by the Siamese network. It is worth noting that the clusters are generally compact which makes them amenable to classical ML techniques for final prediction. Note: The overlap of the clusters is due to the projection of the 20-dimensional embedding into 2D space.

The top-line results of the algorithm are given in **Table 2.** It’s notable that even without specific engineering of the network architecture, the algorithm can accurately predict most of the proper SDTM mappings. In most of the cases, the nearest neighbor is a good predictor of a given class. Looking at the embedding in **Fig 3**, it is apparent that the Siamese networks can generate compact embeddings that are amenable for traditional ML techniques to accurately separate classes. Generally, fields that map to the same SDTM field are similar in their embedding space. Thus, it’s not entirely surprising that the end result shows good classification accuracy. It’s important to note that all of these results are reported in the holdout set only. The self-prediction accuracy of the algorithm is greater than 99%. However, issues such as the disjoint nature of the vocabulary between the testing and training sets lead to lower accuracy when looking at the holdout set.

**Table 2:** Overall accuracy and macro F1 scores using 3 methods of evaluating predicted labels with the siamese network along with the two comparison methods that were implemented in prior attempts.

	Overall Accuracy	Macro F1
**Correct class found in top 5 nearest neighbors**	**0.904**	**0.899**
**Correct class predicted by nearest neighbor**	**0.834**	**0.831**
**Correct class is most common of 5 nearest neighbors**	**0.849**	**0.846**
**XGBoost with TF-IDF**	**0.761**	**0.604**
**RoBERTa Class Prediction**	**0.810**	**0.643**

**Fig 4:**
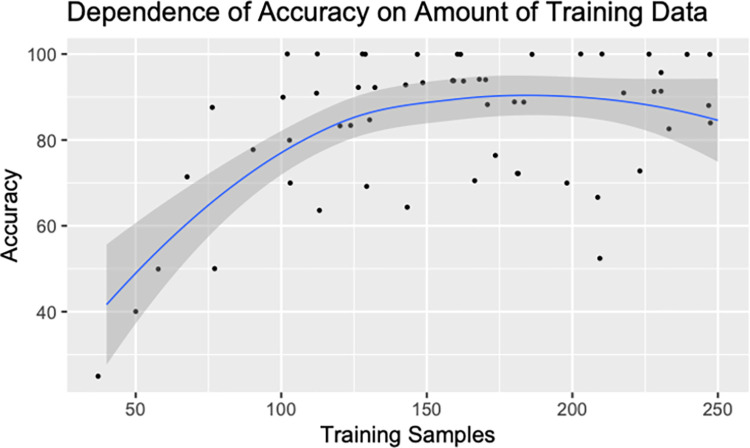
There is a strong dependency with respect to the amount of training data for a specific class with the overall accuracy of the classifier. Because of this dependence, it was determined that utilizing a larger dataset would be more useful than adjusting the network architecture at this point.

Though on average, the accuracy is high, there is a relatively large spread in terms of the accuracy per class. While the majority of the classes exhibit accuracies greater than 90%, the minimum accuracy is 25%. Examining this further in **Fig 4,** there is a clear trend with the amount of training data available vs. the amount of training data available for it. In terms of the fields that are predicted poorly tend to have a low amount of training data associated with them. Therefore, it probably isn’t entirely surprising that for these cases the accuracy is quite low. In all cases, it appears that with fewer than 100 examples within their training data, there isn’t sufficient information to capture the variability of how the field is represented. With greater than 100 training samples, we see good accuracy–often greater than 90% of the fields are predicted properly. Notably, however, the two fields CMDOSFRQ and CMDOSTXT show significant accuracy issues even though they have relatively large amounts of training data.

With the results of the n-nearest neighbor classification, there isn’t a significant difference between using the nearest neighbor or the majority vote of the 5 nearest neighbors. There is a slight improvement using the presence of the proper class, with the majority of the wrong predictions coming from fields with low amounts of training data. Furthermore, many of the fields that are incorrectly mapped appear to be related fields, though appearing in different domains. For instance, AEENDTC (adverse event end date) is in a few cases found to be incorrectly mapped to CMENDTC (concomitant medication end date).

In comparison to the traditional implementations that were evaluated, there was a significant increase in the accuracy of this method even without any specific level of NN architecture tuning (**Table 2**). On average there was a 10 to 15% improvement in the overall classification accuracy when compared to the traditional techniques as defined by the proper labels being predicted. However, the prediction results were more consistent across different classes as evidenced by the significantly higher macro-F1 scores.

## Discussion

The initial hypothesis behind this work was that there was sufficient information in the metadata associated with EDC fields that it was possible to determine the actual SDTM field that they matched to. In this evaluation, it was shown that for the set of domains that the data was trained and tested on this was the case. The use of Siamese networks allows one to generate embeddings such that EDC fields that were mapped to the same SDTM field were embedded close together in the same space. Most of the inaccuracies could be associated with the following issues: the lack of training data for certain domains and the lack of overlapping vocabularies that prevent the embedding layer from generating informative embeddings for some of the training data.

Both of these appear to be data-related issues and could be resolved through the expansion of the dataset. Swapping the input to utilize character-based embedding vs. word level embeddings did not yield better performance. We hypothesize that this is due to the missing vocabulary was generally in the FormOID/FieldOID entries and for fields with few pairs, the character embeddings did not yield any significant information, thus there will be more benefit for expanding the dataset then trying to tune the model for better performance. We have opted to keep a word-based embedding approach under the hypothesis that such an approach would be more amenable to future work on model interpretability.

We hypothesize that the biggest reason for the improvement in accuracy vs. more traditional methods is the use of Siamese networks and the expansion of the amount of data that can be used for training the models. Rather than trying to fit a high dimensional model on a few thousand entries, by utilizing the Siamese network, it’s possible to fit a model on a much larger set of data, and thus maximize the generalizability of the model. However, it is important to note that the system will still benefit from additional data. During the training process of the Siamese network, the within-sample accuracies in terms of predicting similarity were greater than 99.9%, and while the hold-out set showed high levels of accuracy, the gap of roughly 10% is still a gap that can be addressed with more data.

It was observed that there are two primary factors driving the prediction errors. The first categories of errors are common fields that are spread across different domains. For instance, in many cases, different domains will have fields that record dates and times i.e. AEENDTC and CMENDTC and in these cases, they can have very similar metadata, differing only by the FormOIDs and/or FieldOIDs. Thus looking at fields on a case-by-case basis may lead to the misclassification of these fields. The other source of errors can be attributed to the lack of overlap in the vocabulary used between the training and the testing sets. In the two notable examples where there was a significant lack of vocabulary shared, the CMDOSFRQ and CMDOSTXT fields, it was not possible to obtain high-quality embeddings in the testing set.

In the first case, we believe that a two-step process of predicting the actual SDTM domain from all of the metadata used on the form prior to predicting the field may reduce the errors that fall into this category. In the second case, this is a function of the limited vocabulary seen in the training set and is something that can be rectified with more training data. As mentioned earlier, pre-trained embeddings were not found to be directly useful given the amount of abbreviations and jargon that is normally found in the Rave metadata. However alternative architectures that combine a custom embedding layer as well as pre-trained embeddings may allow for improved performance.

The one primary shortcoming with the system as presented is that it only does a 1–1 mapping of EDC fields to their respective SDTM fields. Essentially every field in the EDC is assumed to map to a single SDTM field. However, in many cases, a specific field may end up in multiple SDTM fields. For instance, adverse event forms may capture information about the adverse event as well as the associated medication taken for the adverse event. In such cases, the date field found in that one form can be propagated to multiple SDTM fields. We expect that such cases can be handled by treating these multiple field mappings as their own custom SDTM field i.e. a field that maps to both AESTDTC and CMSTDTC can be instead treated as a field that maps to “AESTDTC/CMSTDTC” to differentiate these cases from the times when the date field maps to only a single AESTDC field. However, this was not investigated further given the small amount of cases that were present in our training dataset.

Another limitation that has not been addressed in this work is the need to predict fields that are the result of calculations. For instance, fields such as BMI are generally not recorded directly but instead are calculated from two separate fields. However, while this problem is not solved in this work, this work represents an important step in the process. This is because once standardized field names can be generated, it simplifies the automated code generation process greatly. We also envision a time when the description of the composite field coupled with standardized columns will allow for tools like Github Copilot to be used to automatically generate the relevant code for the transformation, and this is an approach that we hope to investigate in future work.

One area that was not explored further was whether alternative architectures for the sub-network would yield better results. This was because during the training process of the Siamese network, the ability to predict whether two entries mapped to the same SDTM domain were reaching accuracies of greater than 99%. Thus, it was hard to explore the impact that alternate subnetwork architectures would have had. For the cases where sufficient training data was present the overall accuracy of the holdout set was greater than 95% which suggested that the model was sufficiently generalized for practical uses. That coupled with the fact that the existing model was relatively accurate on the holdout set even with the issues with vocabulary and lack of samples in certain domains means that expanding the training data would have yielded greater benefits than exploring the space of possible network architectures in the sub-network.

Another area that will require further exploration is how to incorporate model interpretability methods such as attention layers, or Shapley values. This would allow end users to better understand why the system made the decision that it did. However, this is currently challenging because the Siamese network part of the model architecture maximizes the similarity/difference between data points, and does not directly predict a class. This is further complicated by the incorporation of a secondary model in which the input features are converted to a numerical vector that is divorced from the original text used in the input.

## Conclusion

In this work, we have shown that the metadata associated with a given CRF field is sufficient to identify the most appropriate mapping to the SDTM field. Furthermore, this process can be done with a relatively simple Siamese neural network. The use of the Siamese network allows the system to generate compact embeddings that make it relatively easy to classify EDC fields into their final SDTM domains, and due to this, we can get state-of-the-art accuracy in this process. In contrast to our implementation of existing methods, it was possible to see a 10% uplift in overall accuracy, with a 32% improvement in the overall F1-macro score indicating that it works significantly better in cases with fewer training examples. More notable is that this level of performance was achieved without any specific hyperparameter tuning.

This method works despite having a relatively small number of training samples. However, the primary limitation of *this work* but not necessarily the method is that more training data needs to be obtained, both for more fields to map, but also for more examples for some of the poorly predicted SDTM fields. Other avenues of improvement that can be made to this model, however, are to incorporate model interpretability methods so that the predictions come with justifications thus allowing a human-in-the-middle workflow that is both more efficient, but also more forgiving of prediction errors.

In an expanded form with more fields and more training data, this offers the possibility to automatically map data in EDC systems to SDTM with minimal human intervention and allows us to remove one of the barriers in clinical research, specifically the time-consuming process it takes to standardize and map data to SDTM.
